# Amorphous BN-Based Synaptic Device with High Performance in Neuromorphic Computing

**DOI:** 10.3390/ma16206698

**Published:** 2023-10-15

**Authors:** Juyeong Pyo, Junwon Jang, Dongyeol Ju, Subaek Lee, Wonbo Shim, Sungjun Kim

**Affiliations:** 1 Division of Electronics and Electrical Engineering, Dongguk University, Seoul 04620, Republic of Korea; 2 Department of Electrical and Information Engineering, Seoul National University of Science and Technology, Seoul 01811, Republic of Korea

**Keywords:** neuromorphic system, memristor, synaptic device, resistive switching, amorphous boron nitride

## Abstract

The von Neumann architecture has faced challenges requiring high-fulfillment levels due to the performance gap between its processor and memory. Among the numerous resistive-switching random-access memories, the properties of hexagonal boron nitride (BN) have been extensively reported, but those of amorphous BN have been insufficiently explored for memory applications. Herein, we fabricated a Pt/BN/TiN device utilizing the resistive switching mechanism to achieve synaptic characteristics in a neuromorphic system. The switching mechanism is investigated based on the I–V curves. Utilizing these characteristics, we optimize the potentiation and depression to mimic the biological synapse. In artificial neural networks, high-recognition rates are achieved using linear conductance updates in a memristor device. The short-term memory characteristics are investigated in depression by controlling the conductance level and time interval.

## 1. Introduction

The recent advances in the Internet of Things (IoT) and artificial intelligence (AI) have led to increased information influx, thus necessitating higher-performing processors, minimal power consumption, and increased computation speeds [1,2]. However, conventional computing systems, based on the von Neumann architectural design have bottlenecks attributed to their serial data processing structures [3,4,5]. In response to this challenge, a recent study proposed an alternative architecture to that used in von Neumann systems [6,7]. A neuromorphic system features parallel networks comprised of neurons and synapses. It performs complex tasks, such as pattern recognition, very efficiently because the processor and memory are closer within a single semiconductor chip compared with their arrangement in the traditional computing system [8].

Next-generation memories, such as phase-change memory (PRAM) [9,10], ferroelectric random-access memory (FRAM) [11,12,13], and resistive-switching random-access memory (RRAM) are commonly employed in neuromorphic systems [14,15,16]. The use of numerous materials in the construction of the resistor imparts different characteristics to RRAM, with each material having different electrical and chemical properties as well as ion-migration behavior. Consequently, RRAM chooses resistive switching layers and metal electrodes that suit its requirements. RRAM stores data in the low-resistance state (LRS) (or ON state) and high-resistance state (HRS) (or OFF state). The mechanism of RRAM is classified as either a filamentous or nonfilamentary type. A filament-type RRAM has nonuniform set/reset voltages. The nonuniformity of the switching is caused by the random formation and rupture of the conduction filament. Owing to the rapid change in the set process from HRS to LRS, the filament type of the RRAM device was also used with the selector as a synaptic device in neuromorphic systems [17]. Conversely, the nonfilamentary RRAM type possesses bidirectional (gradually increasing) conductance. Therefore, the set/reset voltages can be uniformly obtained [18].

Transition metal oxides (TMOs) are deposited using a variety of processes, including sputtering [19,20,21,22,23,24,25,26]. Recently, nitride-based devices have been studied to create efficient synaptic and memory devices to control accurately conductive paths and metal/semiconductor barriers in these devices [27,28,29]. The amorphous BN thin films have attracted significant attention for memory applications owing to wide-bandgap semiconductors with high-thermal conductivity and chemical stability [30,31]. The integration of BN with RRAM enables the construction of neural network models with a smaller memory footprint and capability for faster inferences [32,33,34]. 

In this study, the potentiation and depression of the Pt/BN/TiN device in neuromorphic computing applications are mimicked using changes in conductance values, especially with multi-level cell (MLC) configurations. The performance as a synaptic device is tested by analyzing Modified National Institute of Standards and Technology database (MNIST) data, thus demonstrating the high potential and promise of the Pt/BN/TiN device as a viable candidate for neuromorphic computing implementation. Potentiation is a synaptic plasticity effect induced by the continuous accumulation of conductance, thus promoting the enhancement of connection strength. Conversely, depression involves constant conductance reduction to weakened synaptic connections [35]. The MNIST, well-known for its handwritten digit dataset, is essential for enabling thorough evaluations of deep-learning and machine-learning models. The collection of MNIST images with handwritten numbers plays an essential role in enabling a comprehensive examination and appraisal of various deep- and machine-learning models [36].

The impact of BN films on the resistive and synaptic characteristics is discussed in this study. Pt serves as the top electrode of the device and TiN as the bottom electrode. The materials used for both electrodes affect the resistive switching window and stability. The study focuses on the remarkable nonfilamentary resistive switching characteristics observed in the Pt/BN/TiN memory device. 

## 2. Materials and Methods

**Figure 1:**
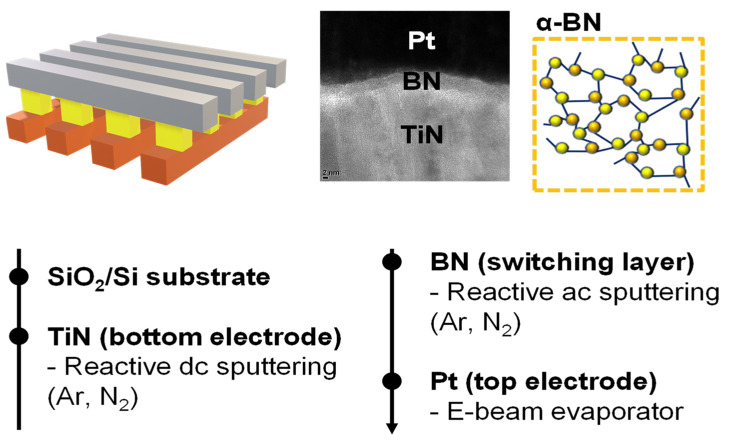
Schematic illustration of Pt/BN/TiN; cross-sectional transmission electron microscopy image of the Pt/a-BN/TiN resistive-switching random-access memory (RRAM) device, and the outline of the fabrication process steps.

The transmission electron microscopy image and process flow of the Pt/BN/TiN device are shown in Figure 1. The bottom electrode comprised a TiN layer (thickness = 100 nm) deposited on a SiO_2_/Si substrate using a reactive sputtering method with a Ti target. During sputtering at room temperature, the Ar flow rate was 19 sccm, N_2_ flow rate was 1 sccm, and the working pressure was 3 mTorr. Subsequently, a BN target was sputtered for a 7 nm BN switching layer. The Ar flow rate was 20 sccm, N_2_ flow rate was 1 sccm, and the working pressure was 3 mTorr. Finally, to deposit the top electrode, Pt 100 nm was grown by e-beam evaporation. All the cells of Pt/BN/TiN were separated using photolithography and a lift-off process. Keithley’s 4200-SCS and 4225-PMU semiconductor parameter modules were respectively used for direct current (DC) sweep and pulse switching to investigate the electrical properties.

## 3. Results and Discussion

**Figure 2:**
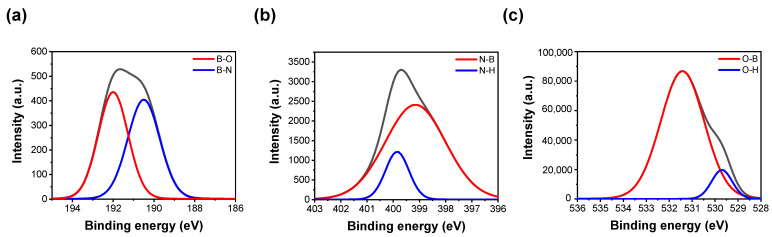
X-ray photoelectron spectroscopy spectra of BN. (**a**) B1s; (**b**) N1s; (**c**) O1s.

X-ray photoelectron spectroscopy (XPS) is a valuable tool used for the analysis of the chemical compositions and properties of target materials. We utilized the XPS in surface mode to investigate the amorphous BN (a-BN) film. In the case of BN, like AlN, it oxidizes easily. Therefore, when deposited using sputter techniques, it is unavoidable to encounter oxygen in XPS analysis [37,38]. Figure 2a displays the B 1s spectra of a-BN that reveal two peaks related to the B-N (190.5 eV) and B-O (192 eV) bonds [39,40]. This suggests that oxygen participates in the a-BN bonding during the deposition of the metal electrode as well as the dielectric. Figure 2b illustrates the N 1s spectra, which have two peaks at 399.15 and 399.85 eV. They are assigned to N-B and N-H, respectively. The O 1s spectra, as shown in Figure 2c, show peaks at 529.7 eV (related to the OH groups) and 531.4 eV (related to the O-B bonds). The oxygen element did not originate from natural oxidation, but from residual oxygen gas inside the equipment during BN sputtering, ultimately indicating the formation of a BNO film.

**Figure 3:**
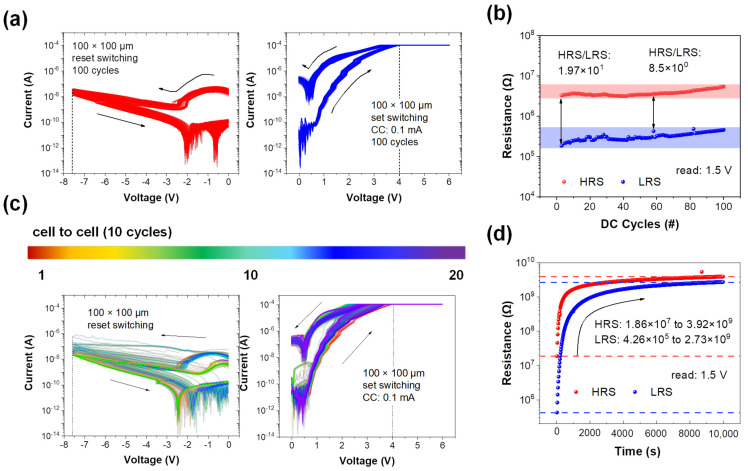
(**a**) One hundred direct current (DC) switching cycles of operation in the Pt/BN/TiN RRAM cell. (**b**) Representative endurance properties of RRAM devices based on the DC operation method. (**c**) Twenty DC cell−to−cell switching cycles of RRAM cells. (**d**) Retention properties of RRAM devices.

To characterize the electrical properties of the Pt/BN/TiN device, the I–V curves were constructed. The I–V curves with different sizes of BN cells are shown for 100 × 100 μm^2^ (Figure 3a), 50 × 50 μm^2^ (Figure S1a), 30 × 30 μm^2^ (Figure S1b), and 10 × 10 μm^2^ (Figure S1c). The entire process was conducted in the minimum compliance current range to prevent the breakdown of the device. Moreover, in the positive voltage range, a set switching occurred from the HRS to the LRS, whereas in the negative voltage range, a reset switching occurred from the LRS to HRS, thus demonstrating typical bipolar switching behavior.

The cell in Figure 3c switches at 4 V with a compliance current (CC) of 0.1 mA and an applied voltage to the cells of −8 V to return to the HRS. For 100 cycles, there is virtually no shift when the condition values are fixed. The cell (with an area of 50 × 50 μm^2^) changes to LRS at approximately 3.2 V at a lower CC of 0.01 mA and to the HRS at −5 V. Given its smaller size, this cell works at a lower CC level as there are relatively fewer nitrogen and oxygen ions (vacancies) involved in the electrical switching [41]. This trend is also observed in the cells with areas equal to 30 × 30 μm^2^ and 10 × 10 μm^2^. The cell with an area of 30 × 30 μm^2^ switches using a current of 0.01 mA at 2.7 V in the set and at −2 V in the reset processes. The reduced ions cause a voltage shift, thus leading to variations during cycling [42]. The cell with an area of 10 × 10 μm^2^ operates at an even lower CC of 0.001 mA, but the reset operation occurs only in the first cycle. Moreover, the endurance deteriorates further compared with the cell with an area of 30 × 30 μm^2^.

**Figure 4:**
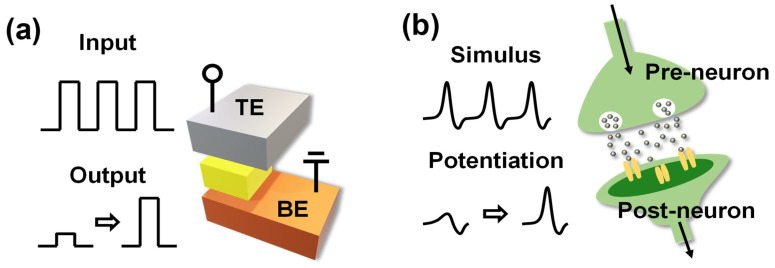
Schematic illustration of similarity between (**a**) RRAM device structure and (**b**) synapse in a neural network.

Figure 3b shows the variation of the resistance range (difference between the HRS and LRS) for 100 cycles. The on/off ratio varied from 19.7 to 8.5. Figure 3c shows the I–V curves for 20 cell−to−cell switching cycles. Randomly selected 20 cells were measured under the same measurement conditions (set: ~4 V, reset: −8 V, and current range: ~0.1 mA). There are no major malfunctions during operation (over 10 cycles and in each cell). Figure 3d shows the change in resistance over time that demonstrates the retention of the device. Over a period of 10,000 s, both HRS and LRS read at 1.5 V increase from 1.86 × 10^7^ to 3.92 × 10^9^ and 4.26 × 10^5^ to 2.53 × 10^9^, respectively. This indicates that the cell with an area of 100 × 100 μm^2^ also exhibits short-term memory characteristics. The BN-based memristor used as a synaptic device emulates the connection between neurons, as shown in Figure 4a. When a stimulus is transmitted from a presynaptic neuron to a postsynaptic neuron, the synapse is involved wherein the neurotransmitter is released [43]. Similarly, the memristor device transfers the signal from the top electrode (Pt) to the bottom electrode (TiN) via the BN layer. Nitrogen ions and nitrogen vacancies that coexist in BN layer due to the presence of anti-Frenkel pairs could affect resistive switching [44,45]. Figure S2 illustrates the multilevel characteristics achieved by varying the compliance current and reset voltage. Applying a fixed set voltage of 6 V while varying the compliance current from 50 μA to 1 mA results in distinct LRS. This outcome is attributed to an increased movement of ions within the BN layer’s interface as the compliance current increases, leading to a higher current flow and the appearance of multilevel characteristics. Additionally, the achievement of multilevel states is demonstrated by increasing the reset voltage. Varying the reset voltage from −7 V to −8.5 V is associated with recombination processes. With an increase in the reset voltage, more recombination occurs with the vacancies, consequently resulting in different HRS. Furthermore, the action of potentiation is achieved, which enhances the weight and weakens depression in the artificial synapse device.

**Figure 5:**
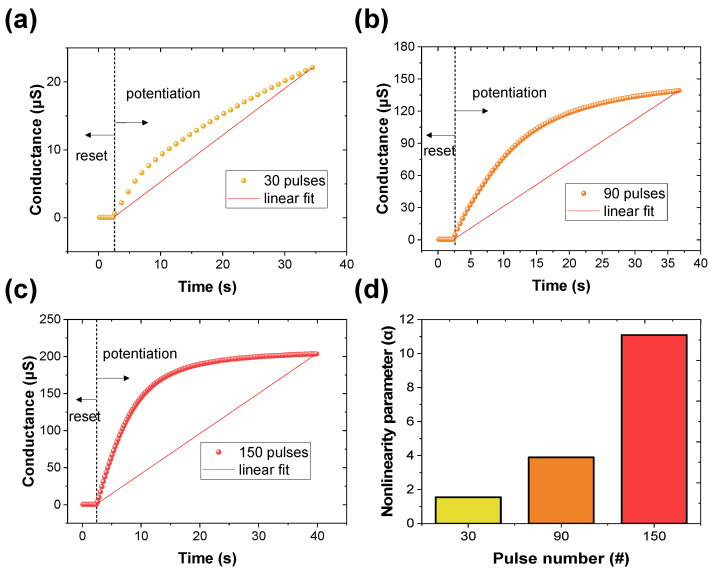
Plots of nonlinear conductance variation when (**a**) 30, (**b**) 90, (**c**) 150 pulses are applied. (**d**) Variation of nonlinearity parameter as a function of the number of pulses.

Linear conductance update is needed in RRAM devices to implement the neuromorphic system. In other words, the nonlinear update of conductance is a challenge as it severely degrades the performance of neuromorphic systems. Unpredictable values during the conductance updating process are not desirable. Thus, the pulse design is tailored to allow potentiation and depression predictions when identical pulses are used. Figure 5a–c illustrates the potentiation performed by setting differently only the number of pulses and interval time within a period of 10 s (as detailed in Figure S3). All data are reset to conductance based on an initializing process before potentiation that involves 10 inputs at −5 V and 50 ms each. The nonlinearity parameter (α) is calculated using the following formula and is depicted in Figure 5d [46].

(1) G={((GLRSα−GHRSα)×w+GHRSα)1α if α≠0,GHRS×(GLRS/GHRS)α if α=0.

where *G_LRS_* and *G_HRS_* are the minimum and maximum conductance, respectively, α is a parameter that attains linearity and symmetry, and w is a synaptic weight increased or decreased according to the pulse (from zero to one). Inputting more pulses within the same period is equivalent to a faster pulse input. These rapid pulses result in a larger conductance and induce abrupt potentiation. This indicates that the pulse oscillation has an impact on nonlinearity.

**Figure 6:**
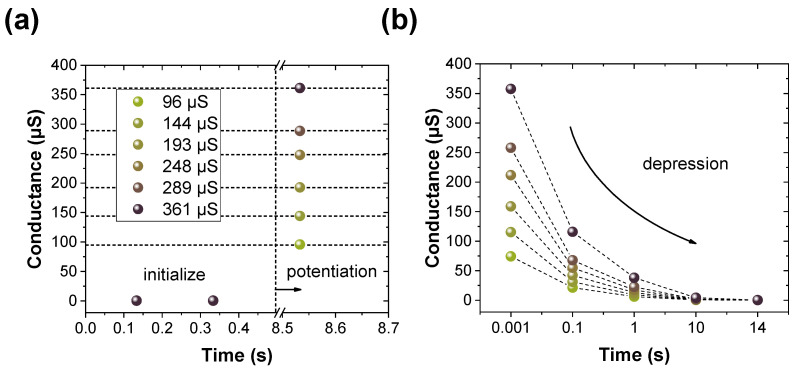
(**a**) Conductance modulation before and after potentiation according to voltage. (**b**) Modulation of depression conductance after potentiation.

**Figure 7:**
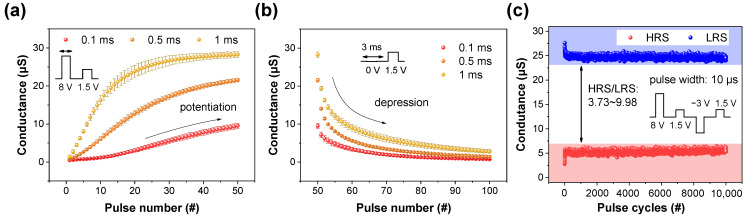
(**a**) Conductance enhancement and (**b**) conductance attenuation responses of six randomly selected cells. (**c**) Endurance behavior of the Pt/BN/TiN RRAM device using the pulse operation method.

For depression, the pulse shown in Figure S4 is transmitted to the device. Like potentiation, after initialization, the conductance is intentionally varied to take values in the range of 100–350 μS, as shown in Figure 6a. Herein, it is only read to suppress dramatic changes, utilizing short-term memory characteristics that are gradually forgotten over time. All the conductance noticeably decreases from 100 ms and become negligibly small after 1 ms. During the period from 10 s to 14 s, these conductance values nearly return to the initial state. Based on this, the decreased conductance can be defined by modulating the interval time between read pulses. The cells are randomly selected and averaged for both potentiation (Figure 7a) and depression (Figure 7b). Unlike the previous approach, the impact of pulse width was investigated. As observed in Figure 7a, an expansion in pulse width corresponds to an increase in both nonlinearity and conductance. This suggests that the pulse width amplifies the change required to reach the next level. A larger conductance demands a longer depression time, as explicitly indicated in Figure 7b. Finally, Figure 7c shows the endurance response of Pt/BN/TiN RRAM devices at the HRS and LRS states after the repetitive application of pulse trains comprising 8 V, 1.5 V, −3 V, and 1.5 V with pulse widths of 10 μs. Throughout the period spanning 10,000 cycles, the HRS and LRS are maintained at values equal to or greater than 3.73. The summary of different types of BN and their applications in inorganic materials is provided in Table 1 [47,48,49,50,51].

Furthermore, to assess the response speed of the devices, we employed SET pulses with a width of 5 ms and amplitudes of 5 V to initiate the resistance state changes, as depicted in Figure S5. Read pulses of 5 ms/3 V were applied both before and after the SET pulses to monitor the resistance switching. Using the pulse pair of 5 ms/5 V, the Pt/BN/TiN device could switch to the LRS within 52.34 µs, while consuming 11.34 nJ of energy during the SET process. This can be calculated by using the formula:

(2) W=V×I×T 

where V is the applied voltage of the pulse, I is the response current, and T is the response time, respectively [52].

**Figure 8:**
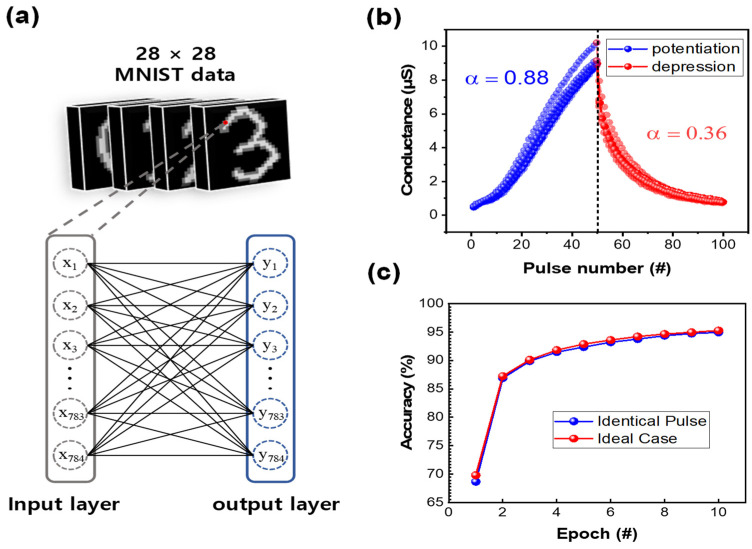
(**a**) Framework for the neural network used in the Modified National Institute of Standards and Technology database (MNIST) simulation of pattern recognition. (**b**) Linearity of five randomly selected cells. (**c**) Accuracy of pattern recognition during 10 successive epochs with identical pulses.

Potentiation and depression are key elements of the software MNIST in artificial neural networks [53,54]. Handwritten numbers, which are sometimes difficult to distinguish, even for humans, cannot be easily recognized by computers. The weight update implemented by the software MNIST and the RRAM hardware make this task even more challenging. When a binary image is input, the system outputs a prediction based on training using the stored dataset for numbers ranging from zero to nine. Each image is represented as a 28 × 28 matrix, where colors closer to white correspond to values closer to 255 in Figure 8a while the color black part converges to zero. These results are used to update the conductance of the RRAM. Finally, the expected value is output following calculations. The potentiation and depression from random cells are depicted in Figure 8b with α being 0.88 and 0.36, respectively. An epoch refers to the number of times the 60,000 images are repeated. When implemented 10 times, the Pt/BN/TiN device achieves a high accuracy equal to 94.96%.

## 4. Conclusions

In this study, we aimed to operate BN-based RRAM as a synaptic device in an artificial neural network. With a Pt/BN/TiN structure, XPS analysis revealed the involvement of boron, nitrogen, and oxygen bonds. To understand the device’s switching mechanism, we used cells with various sizes and adopted the 100 × 100 μm size which yielded minimal variations. We obtained identical I–V curves from 10 randomly selected cells during 100 cycles. Based on the DC switching results, we proceeded with potentiation and depression. For potentiation, we compared linearity by applying multiple pulses within the same period. As the pulse frequency increased, the conductance and nonlinearity increased. For depression, six states were set from 100 to 350 μS, and readings were performed over time for all of them. When XX was operated as hardware for MNIST using the optimal values, we achieved a high-recognition rate equal to 94.96%. This study demonstrated that BN-based RRAM can achieve high performance in neuromorphic systems.

## Figures and Tables

**Table 1:** Comparison of the performance of other BN type materials or inorganic materials.

No	Structure	V_set_/V_reset_	ON/OFF Ratio	Endurance (Cycles)
1	Cu/SiO_2_/ZrO_2_/SiO_2_/TiN	1/−1	10^2^	10^3^
2	ITO/WO_3_/ITO	0.25/−0.42	10^2^	10^8^
3	Ti/AlO_x_/Ti	0.65/−1.15	10^3^	75
4	Pt/ZnO/IZO	2.5/−2	9.12 × 10^2^	10^5^
5	Ti/h-BN/CuNi	0.7/−0.5	10^4^	10^2^
6	Pt/a-BN/TiN	4/−8	1.97 × 10^1^	10^4^ (In this work)

## Data Availability

Not applicable.

## Supplementary Materials

The following supporting information can be downloaded at: https://www.mdpi.com/article/10.3390/ma16206698/s1, Figure S1: I-V curve according to (a) 50 × 50 μm^2^, (b) 30 × 30 μm^2^ and (c) 10 × 10 μm^2^, respectively; Figure S2: Different compliance currents and reset voltages were used to achieve a multilevel state; Figure S3: Pulse scheme for voltage and time interval variations; Figure S4: Pulse scheme for initialization voltage and depression interval time; Figure S5. The switching speed of the SET pulse for Pt/BN/TiN devices is illustrated in the graph. The black lines represent the applied voltage, while the red lines depict the current response pulses.
