# Supplementary material for: Amorphous BN-Based Synaptic Device with High Performance in Neuromorphic Computing
Source: Materials (Basel). 2023 Oct 15;16(20):6698. doi: 10.3390/ma16206698 (PMC10608025; doi:10.3390/ma16206698)
Supplement: Supplementary file 1 [file materials-16-06698-s001.zip › materials-2635733-supplementary.pdf]

materials

Supplementary Materials

Amorphous BN-Based Synaptic Device with High Performance in Neuromorphic Computing

Juyeong Pyo [1,†], Junwon Jang [1,†], Dongyeol Ju [1], Subaek Lee [1], Wonbo Shim [2,*] and Sungjun Kim [1,*]

[1] Division of Electronics and Electrical Engineering, Dongguk University, Seoul 04620, Korea; joozero0908@gmail.com (J.P.); thdn159@gmail.com (J.J.); judongyeol0117@gmail.com (D.J.); leesb981228@gmail.com (S.L.)
[2] Department of Electrical and Information Engineering, Seoul National University of Science and Technology, Seoul 01811, Korea
* Correspondence: wbshim@seoultech.ac.kr (W.S.); sungjun@dongguk.edu (S.K.)
† These authors contributed equally to this work.

Citation: Pyo, J.; Jang, J.; Ju, D.; Lee, S.; Shim, W.; Kim, S. Amorphous BN-Based Synaptic Device with High Performance in Neuromorphic Computing. *Materials* **2023**, *16*, x. https://doi.org/10.3390/xxxxx

Academic Editor(s): Stephan Menzel

Received: 13 September 2023
Revised: 13 October 2023
Accepted: 14 October 2023
Published: date

Copyright: © 2023 by the authors. Submitted for possible open access publication under the terms and conditions of the Creative Commons Attribution (CC BY) license (https://creativecommons.org/licenses/by/4.0/).

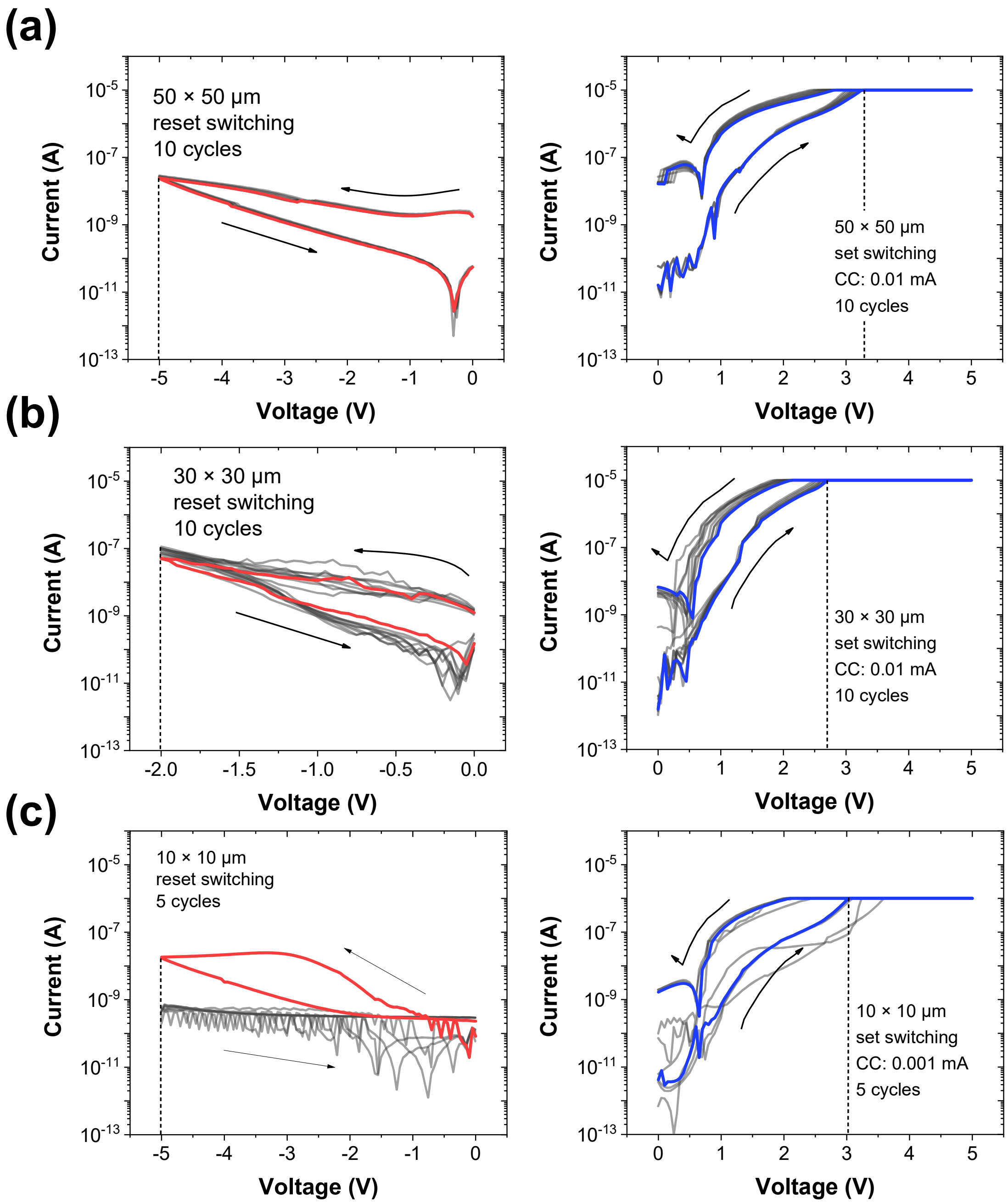

Figure S1. I-V curve according to (**a**) 50 × 50 μm^2, (**b**) 30 × 30 μm^2 and (**c**) 10 × 10 μm^2, respectively.

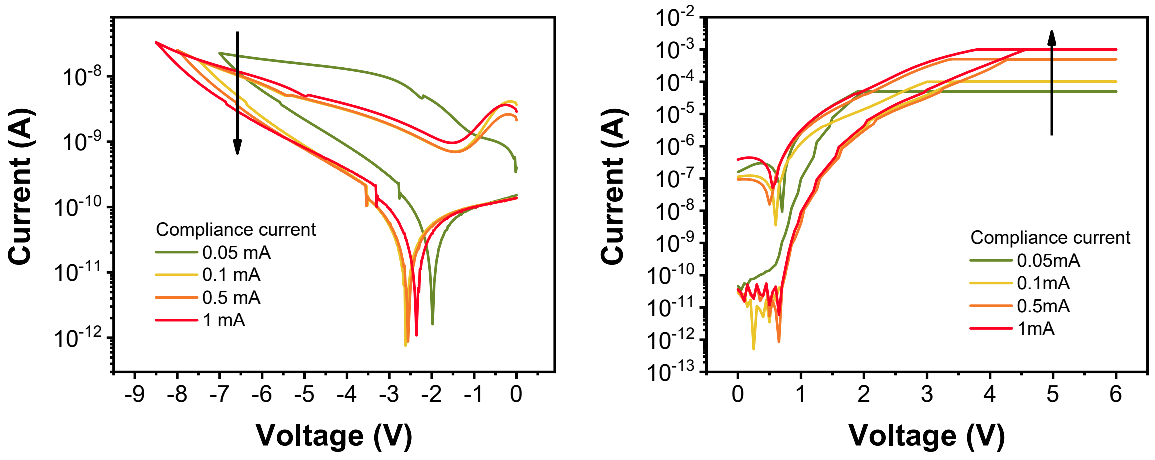

Figure S2. Different compliance currents and reset voltages were used to achieve a multilevel state.

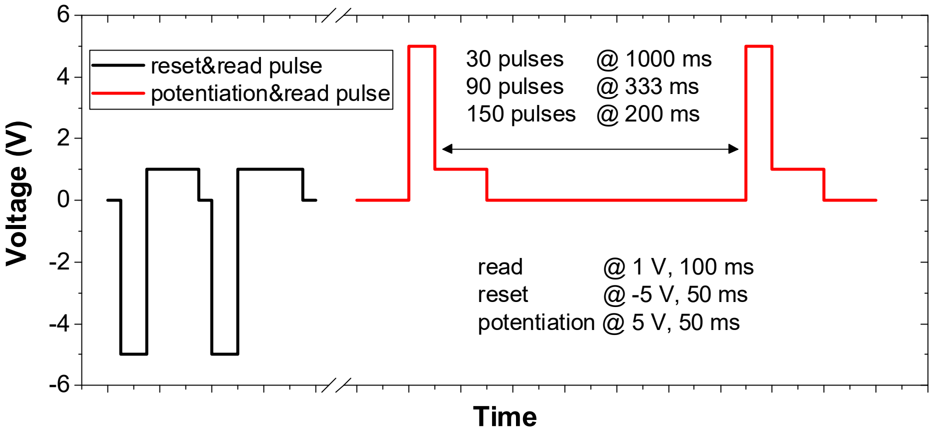

Figure S3. Pulse scheme for voltage and time interval variations.

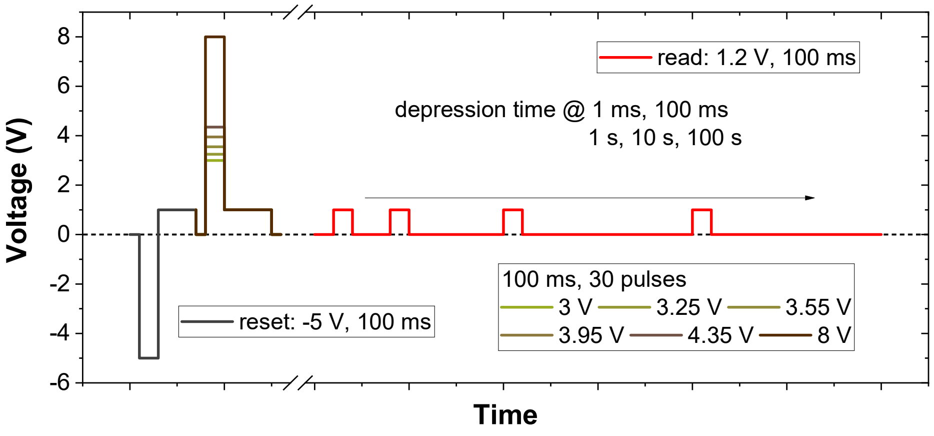

Figure S4. Pulse scheme for initialization voltage and depression interval time.

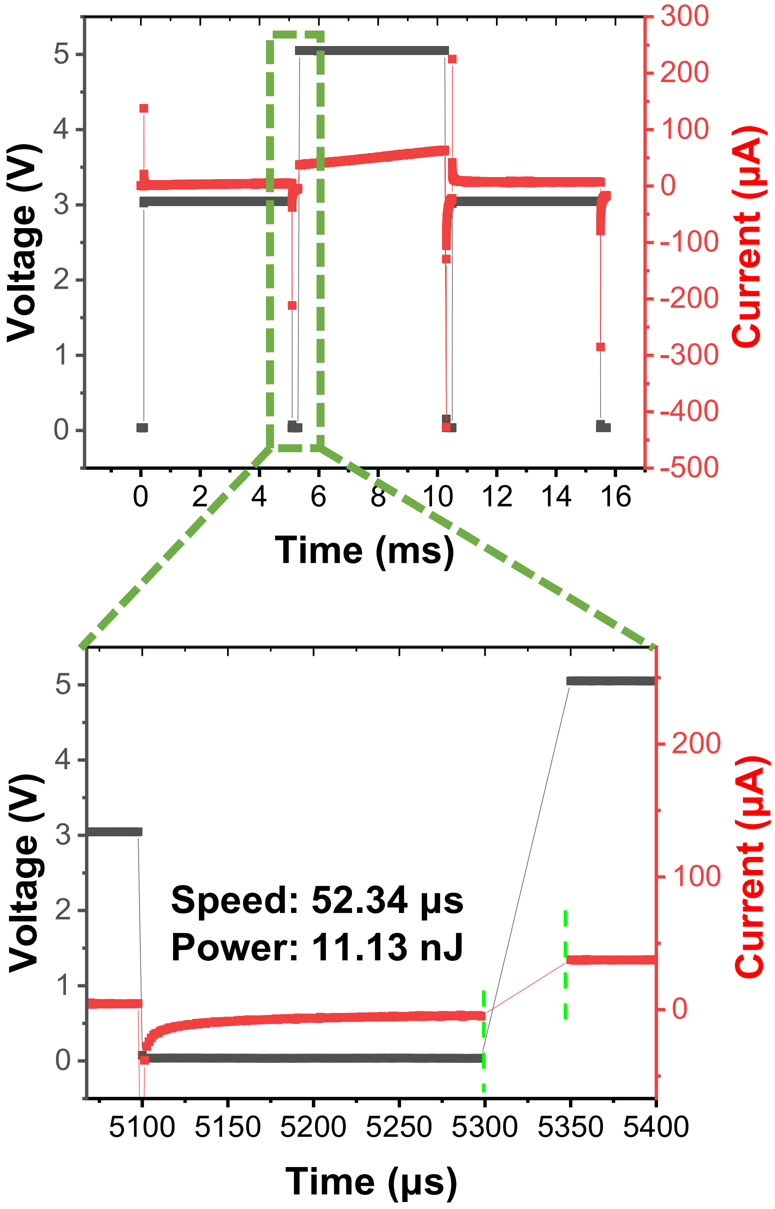

Figure S5. The switching speed of the SET pulse for Pt/BN/TiN devices is illustrated in the graph. The black lines represent the applied voltage, while the red lines depict the current response pulses.
